# A simplified strategy for stent fixation using a defect-closure system in refractory esophageal stricture

**DOI:** 10.1055/a-2771-4378

**Published:** 2026-01-20

**Authors:** Marco Spadaccini, Davide Massimi, Giacomo Marcozzi, Matteo Colombo, Alessandro Fugazza, Roberto De Sire, Alessandro Repici

**Affiliations:** 1 9268 Department of Gastroenterology, IRCCS Humanitas Research Hospital, Rozzano, Milan, Italy; 2 437807 Department of Biomedical Sciences, Humanitas University, Pieve Emanuele, Milan, Italy; 3 9307 IBD Unit, Department of Clinical Medicine and Surgery, Federico II University Hospital, Naples, Italy

Refractory esophageal strictures due to benign conditions (such as post-radiation therapy, endoscopic resection, or caustic ingestion) are often managed with endoscopic stenting. Despite its effectiveness, stent migration remains a major limitation, with studies reporting rates of up to 40% 1 . To address this, stent fixation strategies have been developed 2 . An X-Tack defect closure system (Boston Scientific, Marlborough, MA, USA), originally designed for closure of gastrointestinal defects, has also been used for stent anchoring 3 4 . Conventionally, the device utilizes four helical tacks, a running suture, and a cinch. We describe a simplified approach using tack-only fixation, omitting the suture to make the procedure more straightforward.

**Fig. 1:**
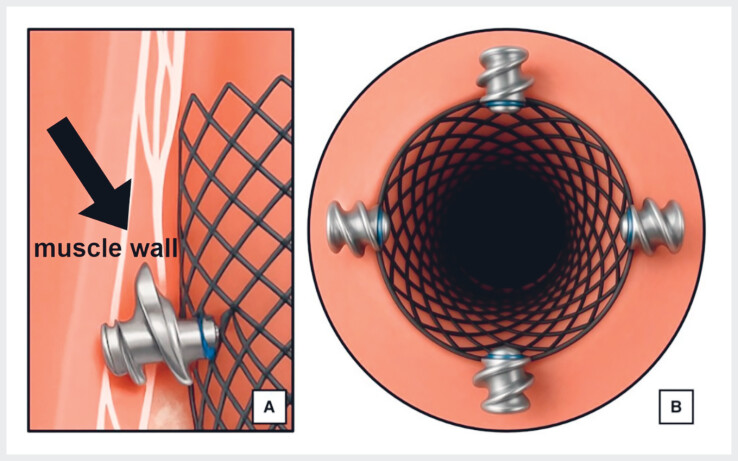
Tacks were advanced through the stent mesh into the healthy apposing esophageal tissue.

A 19-year-old patient with caustic-induced esophageal strictures, refractory to multiple dilations, underwent placement of a fully covered stent. Unfortunately, the stent migrated after 2 days and was removed. A second stent was placed and secured using the X-Tack system. After confirming correct positioning by free contrast flow into the stomach, the polypropylene suture was cut and each tack was loaded individually onto the tack-driver catheter. The first tack was advanced through the stent mesh into healthy apposing esophageal tissues ( Fig. 1 ). Secure fixation was verified by the gentle traction of the device prior to release. Additional tacks were deployed in distinct areas along the proximal stent edge, ensuring stable anchorage ( Video 1 ).

A simplified tack-only approach was adopted for stent fixation in a caustic-induced esophageal stricture. Tack placement along the proximal stent edge achieved secure and stable anchoring.Video 1

Four weeks later, the stent was removed, with endoscopy showing significant improvement of the stricture. The patient has since tolerated a soft diet without further intervention.

This tack-only fixation technique stabilized the stent effectively, prevented migration and simplified the procedure. By eliminating the suture component, the method offers a user-friendly and reproducible approach, potentially extending the use of the X-Tack system even to endoscopists less familiar with defect closure applications.

Endoscopy_UCTN_Code_TTT_1AO_2AZ
